# Preparation and Characterization of a Novel Tragacanth Gum/Chitosan/Sr-Nano-Hydroxyapatite Composite Membrane

**DOI:** 10.3390/polym15132942

**Published:** 2023-07-04

**Authors:** Shuo Tang, Liuyun Jiang, Zhihong Jiang, Yingjun Ma, Yan Zhang, Shengpei Su

**Affiliations:** 1 National & Local Joint Engineering Laboratory for New Petro-Chemical Materials and Fine Utilization of Resources, College of Chemistry and Chemical Engineering, Hunan Normal University, Changsha 410081, China; 2 Key Laboratory of Light Energy Conversion Materials of Hunan Province College, Hunan Normal University, Changsha 410081, China

**Keywords:** tragacanth gum, chitosan, nano-hydroxyapatite, ionic crosslinking, composite membrane

## Abstract

It is a great challenge to obtain an ideal guided bone regeneration (GBR) membrane. In this study, tragacanth gum (GT) was introduced into a chitosan/nano-hydroxyapatite (CS/n-HA) system. The effects of different component ratios and strontium-doped nano-hydroxyapatite (Sr-HA) on the physical-chemical properties and degradation behavior of the CS/Sr-n-HA/GT ternary composite membrane were investigated using Fourier transform infrared spectroscopy (FT-IR), X-ray diffraction (XRD), scanning electron microscopy (SEM), contact angle, electromechanical universal tester and in vitro soaking in simulated body fluid (SBF). The results showed that CS could be ionically crosslinked with GT through electrostatic interaction, and Sr-n-HA was loaded via hydrogen bond, which endowed the GT/CS/n-HA composite membrane with good tensile strength and hydrophilicity. In addition, the results of immersion in SBF in vitro showed that CS/n-HA/GT composite membranes had different degradation rates and good apatite deposition by investigating the changes in pH value, weight loss, water absorption ratio, SEM morphology observation and tensile strength reduction. All results revealed that the CS/Sr-n-HA/GT (6:2:2) ternary composite membrane possessed the strongest ionic crosslinking of GT and CS, which was expected to obtain more satisfactory GBR membranes, and this study will provide new applications of GT in the field of biomedical membranes.

## 1. Introduction

Guided bone regeneration (GBR) membranes play a crucial role in accelerating bone defect repair, which is covered in the defect region to prevent epithelial cells and fibro-blasts from growing into the defect, and creating an isolated space for bone cells growth under the barrier membrane [1,2]. The ideal GBR membranes should possess high mechanical strength and good bio-compatibility, osteoinductivity and biodegradability [3,4]. Chitosan (CS) is a glucosamine-deacetylated product of chitin, which could promote various cells’ adhesion and proliferation because of its similar structure to the extracellular matrix (ECM) of component glycosaminoglycans, so it has been widely used in the biomedical field [5,6,7]. However, pure CS membrane lacks osteoconductivity for guided bone tissue regeneration. To improve the osteogenic effectiveness, nano-hydroxyapatite (n-HA) is usually chosen to obtain the n-HA/CS composite membrane because n-HA has osteoconductivity and osteinductivity due to its similar chemical composition with the natural bone [8,9,10]. Unfortunately, the poor mechanical properties of the n-HA/CS composite were expected to increase, and the fast degradation needs to be slowed down. It was confirmed that the introduction of poly-anion electrolytes could enhance the mechanical properties via ionic cross-linking, which avoids toxic chemical cross-linking [11,12,13]. In our previous study, we also reported related research on n-HA/CS-based composite membranes utilizing carboxylated bamboo fiber or carboxymethyl cellulose [14,15]. To explore more novel composite membranes, some other natural poly-anion polymers have potential to be used to make similar membranes, such as sodium alginate, Xanthan gum and Gellan Gum.

Tragacanth gum (GT) is a natural anionic polysaccharide, which is a kind of colloid secretion from the British plant Astragalus. It has good bio-compatibility, biodegradability, non-toxicity and acid resistance, and it has been widely used in food additives, thickeners, stabilizers and hydrogels [16,17,18]. Moreover, tragacanth gum has also been used in drug carriers, wound healing dressings and skin regeneration [19,20,21]. It has been reported in the preparation of nanofibers used as antibacterial wound dressings using ethyl cellulose containing honey and tragacanth gum as raw materials. The results indicated that tragacanth gum had good antibacterial properties, and the prepared nanofibers could be used for wound repair [22]. However, there has been no report on whether CS could be ionically cross-linked with GT to prepare a CS/n-HA/GT composite membrane with high mechanical properties and suitable degradation to meet the GBR membrane, which is worth investigating.

Additionally, the biological properties of the traditional n-HA are worse than those of biological apatite owing to the lack of trace elements; for example, fluorine, silicon, magnesium, sodium, etc. Therefore, preparing substituted HA via ion doping has been a popular way to improve its biological properties [23,24,25]. Among them, strontium has aroused people’s interest, and many works in the literature have demonstrated that strontium-substituted HA (Sr-n-HA) is a promising nanoparticle that could be used as components of synthetic bone substitute materials, additives for pharmaceutical preparations or food additives for systemic distribution, and can be potentially used to transport strontium to bone tissue and promote bone regeneration [26,27,28]. Therefore, it is necessary to explore the effect of Sr-n-HA on the CS/GT composite membrane.

Based on this, in the present study, we attempted to investigate the effect of different component ratios and Sr-HA on the physical-chemical properties and degradation behavior of the CS/n-HA/GT ternary composite membrane. Fourier transform infrared spectroscopy (FT-IR), X-ray diffraction (XRD), scanning electron microscopy (SEM), contact angle and electromechanical universal tester were used. Moreover, the in vitro degradation behavior of all the composite membranes soaked in simulated body fluid (SBF) was investigated using the pH value change of the soak solution, weight loss, the water absorption ratio, SEM morphology observation and tensile strength reduction.

## 2. Materials and Methods

### 2.1. Materials and Membrane Preparations

Tragacanth gum (GT) with a viscosity greater than 1000 was purchased from Yuanye Biotechnology Co., Ltd., Shanghai, China. Chitosan (CS) was bought from Jinan Haidebei Marine Bioengineering Co., Ltd.jinan, China, with a deacetylation degree of 90% and molecular weight of 3 × 10^5^. Sr-n-HA, 100–200 nm in length and 50–80 nm in width, was prepared in our laboratory according to our previous paper [29]. All other agents were analytical grade, including acetic acid, NaOH, Ca(NO_3_)_2_·4H_2_O, Na_3_PO_4_·12H_2_O, Sr(NO_3_)_2,_ and the reagents for the preparation of SBF.

An amount of CS powder was dispersed in deionized water, and the dispersed Sr-n-HA or n-HA slurry was added into the CS suspension solution. Then, GT powder was slowly introduced into the above CS/n-HA or CS/Sr-n-HA mixture solution with high-speed stirring for 4 h. Following this, the mixture solution composed of CS, n-HA or Sr-n-HA and GT was poured into a clean and dry glass plate. Finally, the proper amount of acetic acid was sprayed on the gel membrane with a pocket-sized sprayer, and the CS/n-HA/GT or CS/Sr-n-HA/GT composite membranes were obtained after the solvent had been completely volatilized. The component weight ratios of CS/n-HA/GT were 4:2:4 and 5:2:3 and the ratios of CS/Sr-n-HA/GT were 5:2:3 and 6:2:2. Additionally, CS was dissolved in 2% acetic acid with a concentration of 3 wt%, and the dispersed n-HA slurry was added into the CS solution. The CS/n-HA composite membrane (8:2) was obtained using the solution casting method as control.

### 2.2. Structural Characterization

Fourier transformation infrared (FTIR) analysis of the composite membranes was carried out using a Thermo Niclet 670 spectrometer, collecting in the absorbance mode at a wavelength range between 600 and 4000 cm^−1^.

The phase analysis of the composite membranes was performed using X-ray diffraction (XRD) using a Rigaku Corporation X-ray diffractometer at 40 kV and 45 mA with Cu-Kα radiation in the range of 2θ = 10~70° with a scanning speed of 10°/min.

The surface morphologies of the composite membranes were observed with scanning electron microscopic (SEM, S-520, Hitachi, Japan), after being sprayed with gold.

The contact angles of samples were measured with a Rotating drop interfacial tensiometer (TX500TM, Kono, USA). Stick the membrane sample onto the glass slide to ensure a flat surface. Add 5 μL water droplets carefully onto the surface of the sample. Use a camera to capture droplet images and analyze contact angles using Image J software.

### 2.3. Tensile Strength of the Composite Membranes

According to the ASTM D882 specification for tensile tests, composite membranes of uniform thickness was selected and cut into a rectangular with the dimensions of 50 × 5 × 0.1 mm^3^, measured at room temperature using a universal testing machine (CMT6000, Sans, Changchun, China) with the speed of 50 mm/min at 25 °C. Three parallel specimens were used to determine the average value and standard deviations of each sample.

### 2.4. In Vitro Soaking of the Composite Membranes

SBF was prepared to evaluate the in vitro degradation of the composite membranes, and the process was as follows: NaCl (8.035 g), NaHCO_3_ (0.355 g), KCl (0.225 g), K_2_HPO_4_·3H_2_O (0.231 g), MgCl_2_·6H_2_O (0.311 g), HCl (39 mL 1.0 mol/L), CaCl_2_ (0.292 g), Na_2_SO_4_ (0.072 g) and Tris (6.118 g) were added in turn per 1000 mL [30], and the composite membrane samples were put into a sealed propylene tube with 10 mL SBF with strip sizes of 50 × 5 × 0.1 mm^3^ at 37 °C, respectively.

The samples were removed from SBF after being soaked for 2, 4, 8 and 12 weeks, washed with deionized water and the surface water absorbed with filter paper. The original weight, wet weight and dry weight of the sample was noted as W_1,_ W_2_ and W_3_, respectively. The weight loss and water absorption ratio were given as follows [31]:

$$\mathrm{Weight}\;\mathrm{loss}/\%=\frac{W_{1}-W_{3}}{W_{1}}\;\times \;100\%$$

$$\mathrm{Water}\;\mathrm{absorption}/\%=\frac{W_{2}-W_{3}}{W_{3}}\;\times \;100\%$$

Moreover, the surface morphologies of samples soaked for 12 weeks were observed with SEM, and the pH value of the soaking solution was measured with a pH meter. In addition, the tensile strength reduction in the samples after being dried at the room temperature was measured.

### 2.5. Statistical Analysis

All data were given as mean ± standard error of three samples. Statistical analysis of data was performed with one-way analysis of variance. The value of *p* < 0.05 was considered to be statistically significant.

## 3. Results and Discussion

### 3.1. Characterization of the Composite Membranes

#### 3.1.1. FT-IR Analysis

**Figure 1:**
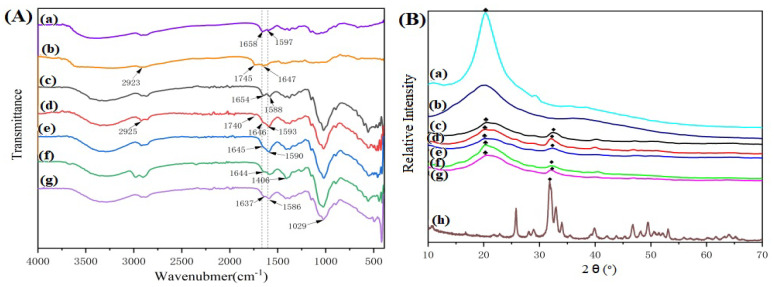
FT-IR (**A**) and XRD (**B**) spectra of samples. (a) CS, (b) GT, (c) CS/n-HA composite membrane (8:2), (d) CS/n-HA/GT composite membrane (4:2:4), (e) CS/n-HA/GT composite membrane (5:2:3), (f) CS/Sr-n-HA/GT composite membrane (4:2:4), (g) CS/Sr-n-HA/GT composite membrane (6:2:2) and (h) n-HA.(The graphic symbols of “◆” and “♣” represent the diffraction peak of n-HA and CS, respectively).

Figure 1A shows the FT-IR spectra of CS, GT and the different CS/n-HA/GT composite membranes. It can be seen that there existed vibrational peaks at 1658 cm^−1^ and 1597 cm^−1^ of CS (Figure 1Aa), which belonged to the amide I and amide II bands, respectively. Figure 1Ab represents the infrared spectrum of GT, and the absorption peaks at 2925 cm^−1^ and 1745 cm^−1^ were the stretching vibration peaks of the C-H bond and the C=O double bond in COO^−^ of GT. Compared with the spectrum of the CS/n-HA composite membrane, 1654 cm^−1^ of CS in the CS/n-HA/GT composite membrane moved to a low wave number, while 1588 cm^−1^ moved to a high wave number, and the peak of GT at 1745 cm^−1^ almost disappeared, indicating that there was an ion cross-linking interaction between CS and GT, forming a poly-electrolyte complex. Comparing the ternary composite membranes with different GT contents, it was found that the CS/Sr-HA/GT(6:2:2) composite membrane had the highest deviation (Figure 1Ag), showing that the ion cross-linking effect of the composite membrane with CS/GT = 3/1 was the strongest, while the deviation of the composite membrane with CS/GT = 4/4 (Figure 1Ad) was the smallest, and there was a weak peak at 1740 cm^−1^, suggesting that excessive GT was not conducive to ion cross-linking. For the composite membranes after the introduction of n-HA and Sr HA (Figure 1(Ae,f)), the position and strength of the peaks were almost unchanged, indicating that the introduced nanoparticles were loaded in the poly-electrolyte of CS-GT, and the strontium-doped n-HA would not have a significant impact on the infrared spectrum of the CS/n-HA/GT composite membrane, which was consistent with the research results in the literature [32].

#### 3.1.2. XRD Analysis

To further understand the interaction among the GT, CS and n-HA, Figure 1B shows the XRD patterns of n-HA, CS, GT and CS/n-HA/GT composite membrane with different weight ratios. The graphic symbols of “◆” and “♣” represent the diffraction peak of n-HA and CS, respectively. For the XRD patterns of CS/n-HA/GT composite membranes, the diffraction peak at 20^°^ was significantly weakened, and the amorphous wide peak of GT was also weakened, compared with those of pure CS and GT, indicating that there was a certain ion cross-linking effect between GT and CS, resulting in a change in the original crystalline state. In different CS/GT systems, the Figure 1Bg composite membrane exhibited the highest peak intensity, suggesting that the ion cross-linking effect of the three components of the CS/Sr-HA/GT (6:2:2) composite membrane was the strongest. Similarly, due to the relatively low content of n-HA (20% wt) in the composite membranes, the characteristic peak intensity at 31° of the traditional n-HA was also weak, proclaiming an interaction between CS, GT and n-HA. In addition, by comparing Figure 1Be with Figure 1Bf, it was found that there was no significant difference in the characteristic peak, which was in agreement with the results of the infrared spectroscopy analysis.

Based on the above results, we supposed that the interaction mechanism of GT, CS and n-HA was that CS was ionically cross-linked with GT via electrostatic interaction, because of their opposite charge, and the three-dimensional poly-electrolyte structure was formed, where n-HA or Sr-HA was loaded in the poly-electrolyte structure via hydrogen bonding, which would endow the membrane with better mechanical properties.

#### 3.1.3. SEM Observation

**Figure 2:**
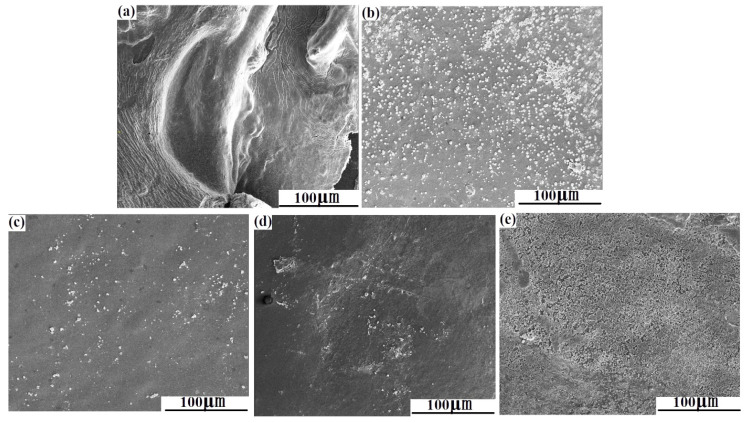
SEM micrographs of composite membrane. (**a**) CS/n-HA (8:2), (**b**) CS/n-HA/GT (4:2:4), (**c**) CS/n-HA/GT (5:2:3), (**d**) CS/Sr-n-HA/GT (4:2:4) and (**e**) CS/Sr-n-HA/GT (6:2:2).

Figure 2 gives the morphologies of CS/n-HA and CS/n-HA/GT composite membranes with different weight ratios. It can be seen that there was an obvious agglomeration of n-HA, and the CS/n-HA composite membrane was full of bumps and holes (Figure 2a). When GT was added, there was little agglomeration of the n-HA particles in the composite membrane, and the interface between n-HA particles and CS-GT matrix was good, which might have originated from the fact that CS could be ionically cross-linked by GT to form the three-dimensional poly-electrolyte structure, which would create a place to load nanoparticles so as to prevent n-HA particles from agglomerating. Comparing the CS/n-HA/GT composite membranes with different weight ratios, there was no obvious difference. However, the CS/Sr-HA/GT (5:2:3) had a slight flatness (Figure 2c), and the CS/Sr-HA/GT composite membrane (6:2:2) displayed the best dispersion and compact interface (Figure 2e), which would enhance the composite membrane with better mechanical property than CS/n-HA composite membrane.

#### 3.1.4. Tensile Strength Test

**Figure 3:**
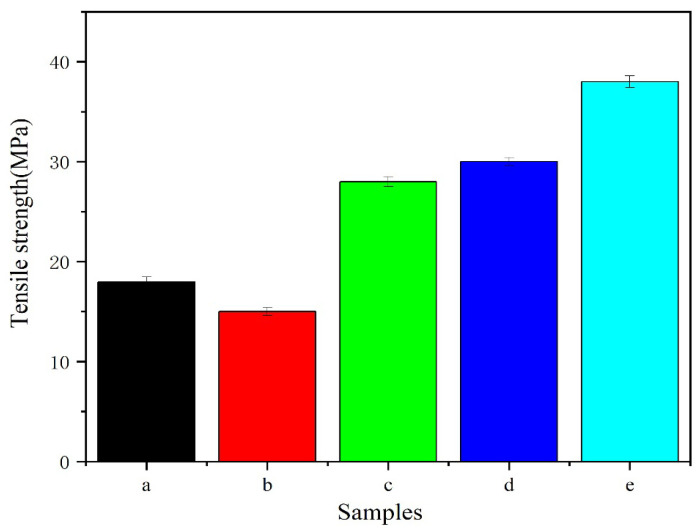
Tensile strengths of composite membranes. (a) CS/n-HA (8:2), (b) CS/n-HA/GT (4:2:4), (c) CS/n-HA/GT (5:2:3), (d) CS/Sr-HA/GT (5:2:3), and (e) CS/Sr-n-HA/GT (6:2:2).

As we know, the initial mechanical properties are important evaluating indicators for GBR membranes. Figure 3 shows the tensile strengths of CS/n-HA and CS/n-HA/GT composite membranes. Obviously, according to the data of mechanical experiments, the tensile strength of CS/n-HA/GT composite membranes was significantly improved compared to the CS/n-HA membrane, which was attributed to the ionic cross-linking of GT with CS. Comparing Figure 3c with Figure 3d, it was found that there was no remarkable difference, showing that the doping of strontium ions in n-HA would not affect the tensile properties of the composite membrane. This was consistent with the analysis results of IR, XRD spectra and SEM observation. However, the tensile strength of the CS/n-HA/GT (4:2:4) composite membrane (Figure 3b) was lower than that of the CS/n-HA membrane, which might be caused by the incomplete ion cross-linking because the higher amount of GT was dispersed irregularly, and the thickness distribution of the membrane was slightly different. When the GT content was gradually reduced, the tensile strength was further improved. In particular, the tensile strength of the component ratio of CS/Sr-HA/GT (6:2:2) was the highest (Figure 3e), and increased more than twice, showing the CS/GT (3:1) was the most suitable weight ratio to be ion cross-linked. The conclusion was in accordance with the previous discussion, and the tensile strength could meet the requirements of the GBR membrane [33].

#### 3.1.5. Contact Angle Measurement

**Figure 4:**
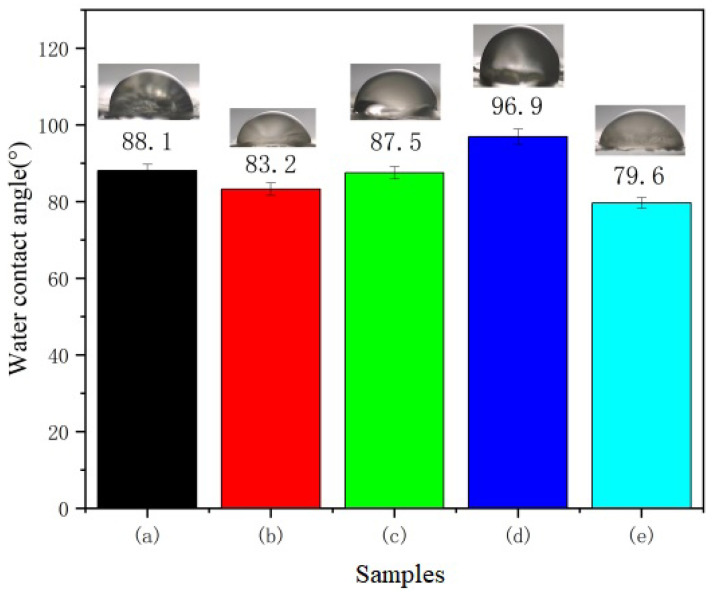
Contact angle of composite membranes. (a) CS/n-HA (8:2), (b) CS/n-HA/GT (4:2:4), (c) CS/n-HA/GT (5:2:3), (d) CS/Sr-n-HA/GT (4:2:4), and (e) CS/Sr-n-HA/GT (6:2:2).

To further clarify the hydrophilicity of the CS/n-HA/GT composite membrane, the contact angle of the membranes are shown in Figure 4. As expected, it can be observed that the contact angle of the membrane would be affected by the introduction of GT and content changes. Firstly, the contact angle of the CS/n-HA composite membrane was close to 90 ^0^ because of the hydrophobic backbone of the chitosan chain. When different proportions of GT were introduced, it was found that the contact angle of the membrane was mostly reduced, and the more GT was introduced, the stronger the hydrophilicity. This was mainly because GT itself is a natural hydrophilic polymer containing -COOH and -OH functional groups. However, the contact angle of CS/Sr-HA/GT (5:2:3) slightly increased, which might be attributed to the slight flatness (seen in Figure 2d of the SEM micrographs), which led to the roughness of the membrane surface, thus increasing the contact angle a little. For other CS/n-HA/GT membranes, the membrane surface tended to be smooth due to the cross-linking effect of GT, effectively reducing the contact angle. And the contact angle of CS/Sr-HA/GT (6:2:2) was the lowest, indicating that the strongest cross-linking effect existed there, which was consistent with the previous analysis, and the improved hydrophilicity would be conducive to cell adhesion.

### 3.2. In Vitro Soaking of the Composite Membranes

#### 3.2.1. Weight Loss Ratios of Samples after Degradation

**Figure 5:**
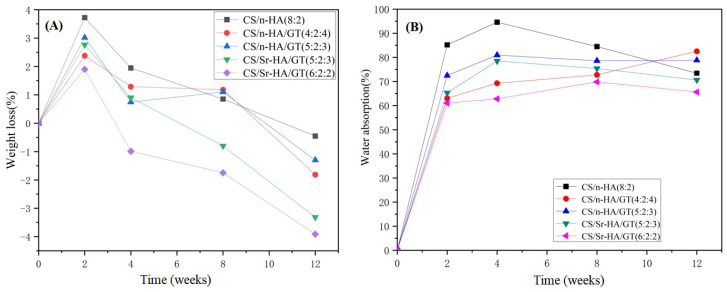
Weight loss (%) (**A**) and water absorption (%) (**B**) of the composite membrane after soaking in SBF for different periods.

Figure 5A shows the weight loss ratios of CS/n-HA and CS/n-HA/GT composite membranes immersed in SBF. Comparing CS/n-HA composite membrane with the samples introduced with GT, it was found that the former overall weight loss ratio was higher than that of other samples, indicating that the CS/n-HA composite membrane had a much quicker degradation rate than the other samples. This was mainly attributed to the ion cross-linking of CS and GT, which could effectively slow down the degradation of the composite membrane. For the CS/n-HA/GT composite membranes, after soaking for 2 weeks, the weight loss ratios of all samples were positive, indicating that the membranes’ weights were decreasing and the composite membranes could be degraded. After 4 weeks of immersion, the weight loss ratios of the CS/Sr-HA/GT (6:2:2) composite membrane were negative, suggesting that the samples’ total weight increased, which was caused by apatite deposition. After 8 weeks, the same happened for the CS/Sr-HA/GT (5:2:3) composite membrane. With the extension of time, at 12 weeks, all composite membranes gradually decreased to a negative value, and the two CS/Sr-HA/GT composite membranes had a greater negative value; that is, the weight increased more, indicating that the apatite deposition amount on its surface was higher than the degradation weight loss, and the reason was that HA was modified by strontium doping, which could improve bio-activity and produce the apatite deposition faster [34]. Moreover, the CS/Sr-HA/GT (6:2:2) composite membranes had the greatest negative value, which was caused by the most apatite deposition and the slowest degradation rate because of the most suitable weight ratio, and the results were in accordance with previous analysis.

#### 3.2.2. Water Absorption Ratios of Samples after Degradation

Figure 5B shows the water absorption ratios of CS/n-HA and CS/n-HA/GT composite membranes. It can be seen that (a) the CS/n-HA composite membrane had the highest water absorption ratio, especially at 2, 4 and 8 weeks, indicating that more cavities existed because of the fastest degradation. After the introduction of GT, the degradation of the CS/n-HA/GT composite membranes was delayed due to ion cross-linking with CS, resulting in a decrease in the overall water absorption ratio of the membrane. For the CS/n-HA/GT composite membranes with different GT contents, the composite membrane of CS/Sr-HA/GT(6:2:2) had the lowest water absorption ratio, which was caused by two reasons. One was that the optimum weight ratio of CS/GT(3:1) brought about the strongest ion cross-linking, which made the composite membrane have the slowest degradation and the least cavities. The other reason was that the introduction of Sr-HA produced more apatite deposition than n-HA, which would cover the cavities and decrease the water absorption ratio. However, the CS/n-HA/GT composite membrane (4:2:4) had the highest water absorption ratio, and the reason mainly originated from the weaker ion cross-linking, causing quicker degradation owing to the inappropriate weight ratio of CS/GT. Moreover, the highest amount of GT had greater water absorption because of the inherent hydrophilicity [35]. The above analysis was consistent with the weight loss ratio results.

#### 3.2.3. SEM Photographs of Samples after Soaking

**Figure 6:**
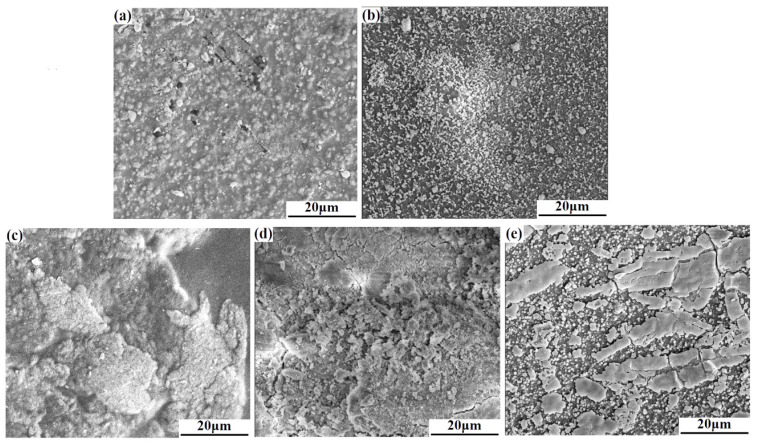
SEM micrographs of the composite membrane after soaking for 12 weeks in SBF. (**a**) CS/n-HA (8:2), (**b**) CS/n-HA/GT (4:2:4), (**c**) CS/n-HA/GT (5:2:3), (**d**) CS/Sr-n-HA/GT (4:2:4) and (**e**) CS/Sr-n-HA/GT (6:2:2).

To further investigate the degradation and the apatite deposition of the composite membrane, Figure 6 gives the SEM photographs of CS/n-HA and CS/n-HA/GT composite membranes soaked for 12 weeks. As expected, it can clearly be seen that there were some cracks on the CS/n-HA composite membrane (Figure 6a), which was the evidence of the membrane degradation. However, when GT was introduced into the CS/n-HA system, little obvious cracks was observed. In addition, it could be found that more new apatite was deposited on the surface of two CS/Sr-HA/GT composite membranes, and the deposition apatite was even connected in pieces on the surface of two CS/Sr-HA/GT(6:2:2) composite membranes (Figure 6e), demonstrating that the introduction of the appropriate proportion of GT could not only delay CS/n-HA composite membrane degradation, but also the Sr-HA would be more conducive for apatite deposition than n-HA, which was in accordance with the result of the weight loss ratio and water absorption ratio. The new deposited apatite might be beneficial for cell attachment and bone formation [36,37].

#### 3.2.4. pH Value Change of Samples during Soaking

**Figure 7:**
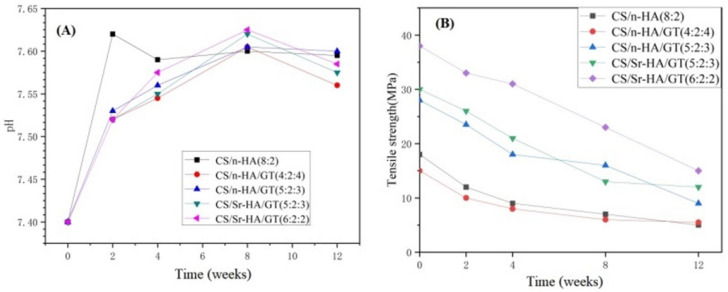
pH value change (**A**) and tensile strength reduction (**B**) of composite membranes after soaking in SBF for different periods.

Figure 7A gives the pH value changes of CS/n-HA and CS/n-HA/GT composite membranes with time. According to the curve, within 0–2 weeks, the overall trend showed an upward trend, as the n-HA on the surface of the composite membrane dissolved and the OH^-^ in the solution increased, resulting in a continuous increase in pH value. However, the pH value of the soaking solution in the CS/n-HA composite membrane increased remarkably due to the faster degradation and greater dissolution of HA. During 2–4 weeks, the pH of the soaking solution of the CS/n-HA composite membrane decreased, because the degradation of chitosan was mainly manifested by the breaking of the 1,4-neneneba glycosidic bond to produce a large number of hydroxyl and carboxyl groups, which led to the pH decrease. However, the pH change tended to be stable owing to the apatite deposition in the late stage of immersion. After the introduction of GT, the pH of the composite membranes remained elevated from 0 to 8 weeks, and the CS/Sr-HA/GT(6:2:2) composite membrane increased the most. This was mainly caused by the fact that the introduction of GT effectively slowed down the degradation of the CS polymers. During 8–12 weeks, the pH decreased slightly because of the weakly acidic production after the dissolution of GT and apatite deposition. Moreover, for the same weight ratio of CS/Sr-HA/GT and CS/n-HA/GT composite membranes, the Sr-HA would produce more apatite deposition, so the pH decreased more. However, the pH value of the CS/n-HA/GT composite membrane (4:2:4) was at the lowest level throughout the entire process, and the reason was that the higher content of GT generated more H^+^ by dissolution. Although there was a subtle difference in the pH of each sample soaking solution, the pH value of all composite membranes had a small change range of 7.40–7.63 during the degradation period, which demonstrated that there was no acid release, and it was suitable for the micro-environment in the human body, so the CS/n-HA/GT composite membranes could meet the basic requirements of harmlessness to the human body [38].

#### 3.2.5. Mechanical Strength Reduction

The mechanical strength of the degradable composite membrane gradually decreased as the soaking time was prolonged because CS and GT are both natural biodegradable polymers. Therefore, it is necessary to study the mechanical strength reduction during the soaking period, which would help to speculate about the in vivo degradation rate. Figure 7B gives the tensile strength reduction in CS/n-HA and CS/n-HA/GT composite membranes in SBF during the entire soaking period of 2, 4, 8, and 12 weeks. According to the data, it could be seen that the tensile strength of all composite membranes gradually decreased during the degradation process. At 0–2 weeks, all membranes underwent rapid degradation, resulting in a significant decrease in strength, and the intensity attenuation of CS/n-HA and CS/n-HA/GT composite membrane (4:2:4) were more significant because CS/n-HA degraded faster without GT, while the CS/n-HA/GT composite membrane (4:2:4) also degraded quickly because the excessive GT made the composite membrane display uneven ion cross-linking. However, in 2–12 weeks, the strength attenuation for most of the membranes slowed down, because the cavity filled by the deposited apatite made the membrane uniform and flat as a whole, thus retarding the strength reduction to a certain extent, while the CS/Sr-HA/GT(6:2:2) composite membrane displayed the largest overall strength attenuation rate, which might have been caused by the introduction of Sr-HA producing more apatite deposition, and the thickness becoming uneven. But at 12 weeks, the CS/Sr-HA/GT(6:2:2) composite membrane still had the highest tensile strength, and CS/n-HA and CS/n-HA/GT composite membranes (4:2:4) had the lowest tensile strength throughout the entire soaking process, indicating that the CS/Sr-HA/GT(6:2:2) composite membrane had the most suitable weight ratio and could meet the supporting function requirements for the GBR membrane.

## 4. Conclusions

To sum up, in this work GT was designed to be introduced into the CS/n-HA system, and the effect of different component ratios and Sr-HA on the ternary composite membrane was investigated. The component weight ratios of CS/n-HA/GT were 4:2:4 and 5:2:3, and for CS/Sr-n-HA/GT they were 5:2:3 and 6:2:2. The main purpose of the study was to explore the probability of preparing a novel composite membrane. The structural characterization confirmed that the CS/n-HA/GT composite membrane could be obtained via ionic cross-linking through the electrostatic interaction of CS and GT, and n-HA was loaded in the poly-electrolyte structure via hydrogen bonding, which endowed the modified CS/n-HA/GT composite membranes with excellent tensile strength, compared to the CS/n-HA composite membrane, especially the CS/Sr-HA/GT (6:2:2) composite membrane, whose tensile strength was improved by over twofold compared to that of the CS/n-HA composite membrane, owing to the most suitable proportion of CS/GT (3:1). Moreover, in vitro simulated body fluid soaking results stated clearly that the CS/n-HA/GT composite membranes had different degradation rates and good apatite deposition. In a word, all results revealed that the CS/Sr-n-HA/GT ternary composite membrane (6:2:2) was the optimal composition membrane, because it possessed higher mechanical properties, more suitable degradation and better bio-activity, and it is expected to develop a more satisfactory GBR membrane. This study will provide new applications for GT in the field of biomedical membranes.

## Data Availability

The data presented in this study are available on request from the corresponding author.
